# Epidemiological Investigation and Phylogenetic Analysis of Major Blood-Derived Pathogens in Sheep from Gansu Province

**DOI:** 10.3390/pathogens15010088

**Published:** 2026-01-14

**Authors:** Jin Luo, Li Ma, Fangyu Xiao, Muhammad Kashif Obaid, Hongfei Zheng, Qiaoyun Ren, Guiquan Guan, Hong Yin, Ping Liu

**Affiliations:** 1 State Key Laboratory of Animal Disease Control and Prevention, Key Laboratory of Veterinary Parasitology of Gansu Province, Lanzhou Veterinary Research Institute, Chinese Academy of Agricultural Sciences, Xujiaping 1, Lanzhou 730046, China; xiaofangyu2024@126.com (F.X.); kashifobaidawkum@gmail.com (M.K.O.); renqiaoyun@caas.cn (Q.R.); guanguiquan@caas.cn (G.G.); yinhong@caas.cn (H.Y.); 2 Animal Husbandry and Veterinary Station in Oula Town, Maqu County, Linxia 747300, China; mqxolxmsyz@163.com; 3 College of Veterinary Medicine, Hunan Agricultural University, Changsha 410128, China; 4 Animal Disease Prevention and Control Center of Gansu Province, Lanzhou 730046, China; 15294202920@163.com; 5 Jiangsu Co-Innovation Center for Prevention and Control of Important Animal Infectious Diseases and Zoonoses, Yangzhou 225009, China

**Keywords:** blood-borne pathogens, epidemiological investigation, phylogenetic analysis, Gansu province, sheep

## Abstract

Investigating the prevalence and molecular genetic characteristics of *Anaplasma ovis*, *Theileria* spp., *Anaplasma phagocytophilum*, and hemotropic *Mycoplasma* infections in sheep populations across different regions of Gansu Province is of significant importance for the prevention and control of these pathogens. A total of 1523 sheep blood samples were collected from 19 counties (districts) in Gansu Province. Pathogen screening was conducted using PCR-based molecular detection techniques, followed by sequencing and phylogenetic analysis of specific genes (e.g., Msp4, 18S rRNA) from selected positive samples. Blood-borne pathogens infections in Gansu Province were widespread but unevenly distributed geographically. *Theileria* spp. and *Anaplasma ovis* were the dominant pathogens, with overall infection rates of approximately 16.7% and 9.6%, respectively. The highest *Anaplasma ovis* infection rate (82.5%) was observed in the Gannan region, where co-infections were common (24/97). An exceptionally high *Theileria* spp. infection rate (87.5%) was detected in the Zagana area. No pathogens were detected in Wuwei, Jingyuan, Huining, Jingtai, Qinghuan, or Maqu. Phylogenetic analysis revealed that the Msp4 gene sequences of *Anaplasma ovis* isolates from Gansu shared 99.48% homology with strains from Europe, Asia, and Africa. *Anaplasma phagocytophilum* isolates also showed high homology (99.53–99.84%) with multiple global strains. Seasonal data indicated significantly higher *Theileria* spp. infection rates in spring (23–34%) compared to other seasons (approximately 12%). Gansu Province is an endemic area for multiple blood-borne pathogens, with distinct regional clustering and seasonality in prevalence. The high conservation of pathogen gene sequences suggests genetic stability. This study provides essential epidemiological baseline data and a scientific foundation for targeted prevention and control of blood-borne pathogen diseases in sheep in Gansu Province.

## 1. Introduction

Blood-borne pathogen diseases represent a core biosecurity threat to global animal husbandry, primarily caused by pathogens such as *Anaplasma* spp., *Theileria* spp., and *Mycoplasma* spp. These pathogens are transmitted through arthropod vectors like ticks and lice. Infection in livestock triggers high fever, hemolytic anemia, progressive emaciation, and multi-organ failure. In severe outbreaks of babesiosis/theileriosis or hemotropic mycoplasmosis, mortality can reach 5–40% in naïve herds [1]. Regionally, lamb mortality attributed to these pathogens in Gansu endemic areas ranges from 15–30% [2]. Globally, blood-borne pathogens in livestock contribute to substantial economic losses, with estimated annual direct costs exceeding $13.9–$18.7 billion for tick-borne diseases [3].

In China, the sheep and goat population exceeds 300 million. Northwestern pastoral regions, characterized by ecological fragility and high vector density, are severely affected by hemoparasitic diseases. Gansu Province serves as an example: designated as a core area in the national meat sheep industry belt, its livestock sector contributes over 35% of its total agricultural output value. However, tick-borne disease outbreaks in recent years have caused lamb mortality rates of 15–30%, directly threatening the livelihoods of millions of farmers and herders, as well as regional economic stability. Notably, Gansu spans three major ecological zones: the Qinghai-Tibet Plateau, the Loess Plateau, and the Mongolian-Xinjiang Desert. Its alternating arid and semi-arid climate provides an ideal breeding ground for vectors like *Haemaphysalis longicornis* and *Dermacentor nuttalli*, further amplifying epidemic risks [4]. Yet, significant gaps persist in understanding the province’s pathogen spectrum, necessitating systematic research to support targeted control strategies.

Although sporadic outbreaks of ovine anaplasmosis have been reported in localized areas of Gansu (e.g., Wenxian and Liangdang counties) [5], existing research suffers from three major limitations. First, survey coverage is fragmented: available data are mostly confined to single counties or cities, lacking systematic sampling across the province’s 14 prefecture-level cities and over 40 pastoral counties. Epidemiological data are particularly scarce for the Longdong Loess Plateau and the Hexi Corridor agro-pastoral ecotone. Second, pathogen spectrum focus is narrow: previous studies predominantly concentrate on single pathogens like *Anaplasma ovis*, overlooking co-infection risks with *Theileria luwenshuni* and *Theileria uilenbergi* and hemotropic mycoplasmas (*Mycoplasma ovis*, formerly *Mycoplasma ovis*). Mixed infections can synergistically worsen clinical symptoms, increasing mortality rates by 2–3 times [6]. Third, molecular characteristics remain unknown: the variation patterns of key genes (e.g., *Anaplasma* Msp4, *Theileria* spp. 18S rRNA) in Gansu strains are unclear, and their genetic evolutionary relationships with domestic and international strains (e.g., Mongolian *A. ovis* isolates, European *B. motasi* vaccine strains) remain unresolved. This severely hinders the evaluation of diagnostic reagents and vaccine suitability. Breakthroughs in molecular biology offer core solutions: designing genus/species-specific primers (targeting genes like Msp4, cox1, 18S rRNA) enables multiplex PCR for simultaneous detection of mixed infections [7,8,9]. Phylogenetic analysis (Maximum Likelihood method) and genetic diversity index calculations (Hd value) based on gene sequences can reveal pathogen population structure, geographic isolation effects, and potential virulence evolution trajectories [10,11,12], providing a molecular epidemiological foundation for regionalized control.

This study employs a stratified random sampling strategy across Gansu’s five major ecological zones (e.g., Gannan Grassland, Longdong Hills, Hexi Oasis), selecting 30 representative sheep farms and collecting 1523 whole blood samples. It aims to address regional control challenges through three objectives: Mapping high-precision epidemiology: Detect infection rates of five core pathogens (*A. ovis*, *A. capra*, *A. phagocytophilum*, *Theileria* spp., *M. ovis*) via nested PCR/real-time PCR, combining GIS spatial analysis to reveal infection hotspots and their correlation with altitude and vegetation index (NDVI). Deciphering mixed infection dynamics: Utilize logistic regression models to quantify pathogen coexistence patterns (e.g., positive synergy between *A. ovis* and *M. ovis*), assessing the dose-effect of mixed infections on clinical indicators like hemoglobin concentration (HGB). Elucidating molecular evolutionary patterns: Sequence target genes from PCR-positive samples (*n* = 254), construct maximum-likelihood phylogenetic trees incorporating 217 global reference sequences, and identify genetic clades of Gansu strains based on 18S rRNA/Msp4 loci. The research outcomes will directly serve three applications: Generating a “Gansu Province Sheep Hemoparasitic Disease Risk Grading Map” to guide targeted tick control and immunization program optimization. Developing a PCR-RFLP rapid typing kit based on Gansu strain-specific SNP sites to enhance field diagnostic efficiency. Providing a scientific basis for cross-border disease transmission warnings in “Belt and Road” livestock trade by comparing genetic distances between Chinese and Central Asian isolates.

## 2. Materials and Methods

### 2.1. Ethics Approval

This study was approved by the Animal Administration and Ethics Committee of Lanzhou Veterinary Research Institute, CAAS (Approval No. LVRIAEC 2025-007).

### 2.2. Study Area and Sample Collection

This province-wide study was conducted across 19 counties/districts in Gansu (March 2024–June 2025), representing all topographic and climatic zones. Sampling employed a stratified random design: ≥5 flocks per county were randomly selected from smallholder (*n* = 5–30), semi-intensive (*n* = 40–96), and intensive (*n* = 100–287) production systems. At each site, peripheral blood samples were aseptically collected from randomly selected sheep in local flocks into EDTA anticoagulant tubes, with source locations recorded. Per flock, ≥30 animals stratified by age (<1 year, 1–3 years, >3 years) and sex were systematically sampled, restricted to clinically healthy animals not recently treated with acaricides/antibiotics. Temporal distribution ensured balanced seasonal coverage (spring: *n* = 246; summer: *n* = 987; autumn: *n* = 290; winter: *n* = 0). All samples were promptly stored at −20 °C for subsequent use. Sample size variation across counties reflected logistical constraints in remote pastoral areas (e.g., Jinchang, Longnan), where sparse sampling (*n* = 5–15) occurred only at geographically isolated farms or sites with restricted veterinary access; these data were retained to preliminarily map pathogen presence but excluded from comparative prevalence analyses.

### 2.3. Genomic DNA Extraction

Genomic DNA was extracted from 200 μL of the blood sample using the Blood Genomic DNA Extraction Kit (Tiangen Biotech, Beijing, China), following the manufacturer’s instructions. The concentration and purity (A260/A280 ratio) of the extracted DNA were measured using a microspectrophotometer (Gene Company Limited, Thermo Scientific, Shanghai, China), and the DNA was stored at −20 °C for subsequent use.

### 2.4. Molecular Detection and Pathogen Identification

Specific PCR methods were used to detect four target pathogens (*Anaplasma ovis*, *Anaplasma phagocytophilum*, *Theileria* spp., and *Mycoplasma* spp.) using published genus/species-specific primers (Table 1). All primers showed no cross-reactivity with related pathogens (e.g., *A. marginale* for *Anaplasma*; *Mycoplasma* for Mycoplasma) in control tests. All PCR reactions included positive controls (plasmid DNA containing target fragments) and negative controls (nuclease-free water). All pathogens’ target gene amplification was conducted with an annealing temperature maintained at 54 °C. PCR products were separated by 1.5% agarose gel electrophoresis, and specific bands of expected sizes were visualized under UV light. Samples with bands matching these sizes were scored as positive.

### 2.5. Gene Sequencing and Phylogenetic Analysis

For genetic evolutionary analysis, target gene fragments from pathogen-positive samples were sequenced and aligned with globally sourced reference strains from GenBank using MEGA 11.0, followed by phylogenetic tree reconstruction through Maximum Likelihood (ML) in MEGA v11 with the Tamura-Nei model (best-fit nucleotide substitution) and 1000 bootstrap replicates, while genetic diversity was quantified via haplotype diversity (Hd), nucleotide diversity (π), and Watterson’s theta (θ) in DnaSP v6, with phylogenetic robustness additionally assessed by Bayesian inference (106 MCMC generations) and nodal reliability measured through bootstrap support values.

### 2.6. Data Analysis

Data analysis was performed using SPSS v28.0. Descriptive analyses calculated pathogen infection rates as: Infection rate (%) = (Number of positive samples/Total samples tested) × 100%, reported with 95% Clopper-Pearson confidence intervals. Inferential analysis compared seasonal differences (spring vs. other seasons) using Pearson’s chi-square test, with statistical significance at *p* < 0.05. This test was justified as: (i) All expected frequencies exceeded 5 (validating chi-square assumptions), (ii) Sample sizes provided >80% power to detect prevalence differences ≥15% (α = 0.05, GPower 3.1).

## 3. Results

### 3.1. Overview of Blood Parasite Infections in Various Regions of Gansu Province

**Figure 1:**
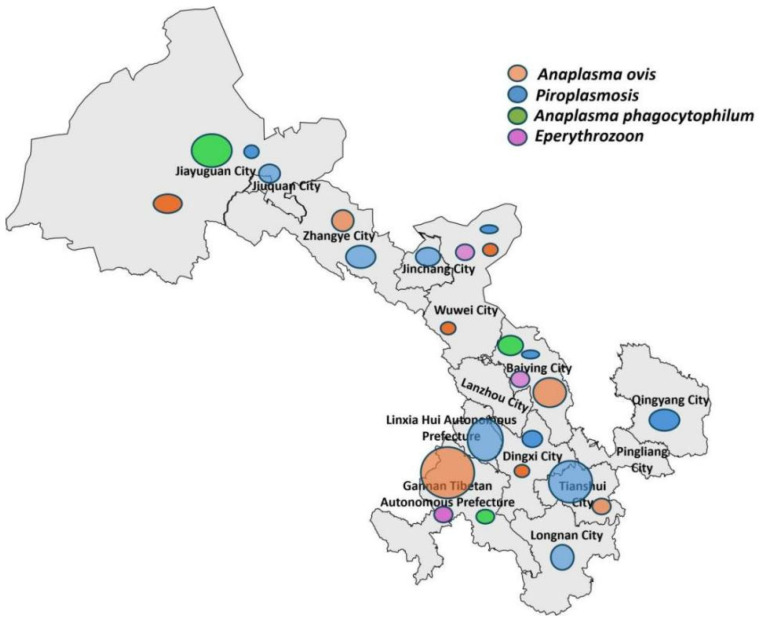
Analysis of the distribution characteristics of blood pathogens in Gansu Province. The size of the marked points indicates different infection levels: the larger the marked points, the more serious the infection, and vice versa.

This study examined 1523 sheep blood samples from 19 counties (districts) in Gansu Province for four types of blood pathogens. The findings indicate that blood pathogen infections in sheep are widely distributed across Gansu Province but exhibit significant geographical variations. Specific detection results are shown in Figure 1. Pathogen screening revealed the following prevalence rates in Gansu province. *Anaplasma ovis* (bacterial rickettsia): 8.7% (95% CI: 7.3–10.3%; *n* = 133); *Anaplasma capra* (bacterial rickettsia): 2.8% (95% CI: 2.0–3.8%; *n* = 42); *Theileria* spp. (apicomplexan parasite): 10.7% (95% CI: 9.2–12.4%; *n* = 163); *Anaplasma phagocytophilum* (bacterial rickettsia): 0.1% (95% CI: 0.0–0.5%; *n* = 2); *Mycoplasma* spp. (bacteria): 0.9% (95% CI: 0.6–1.4%; *n* = 24) (Table 2). No Babesia sequences were identified in PCR-positive samples after sequencing and phylogenetic analysis. Estimates with small sample sizes (*n* ≤ 25) are reported as tentative observations due to limited statistical robustness.

### 3.2. Seasonal Prevalence of Theileria *spp.* Infections in Sheep

**Figure 2:**
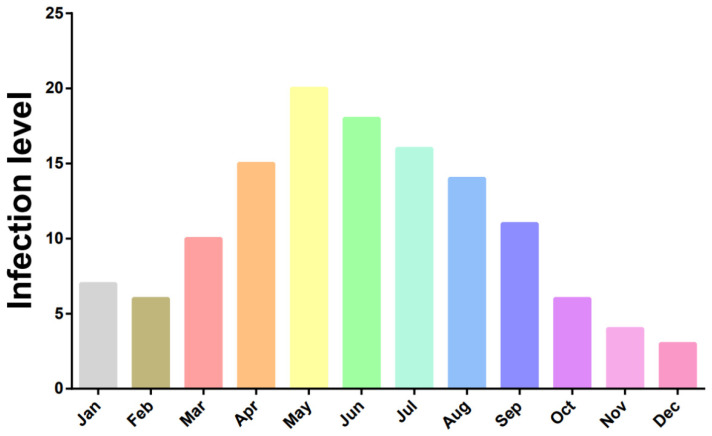
Seasonal prevalence of *Theileria* spp. infections in sheep in different months. Different colors represent different months.

Further analysis of *Theileria* spp. detection data revealed significant seasonal fluctuations in infection rates. Among samples collected during spring, the average infection rate ranged from 23% to 34%. In contrast, samples collected in other seasons (summer, autumn, and winter) showed an average infection rate of approximately 12%. This indicates that spring represents a season of higher risk for *Theileria* spp. infections (Figure 2).

### 3.3. Molecular Characteristics and Phylogenetic Analysis of Pathogens

**Figure 3:**
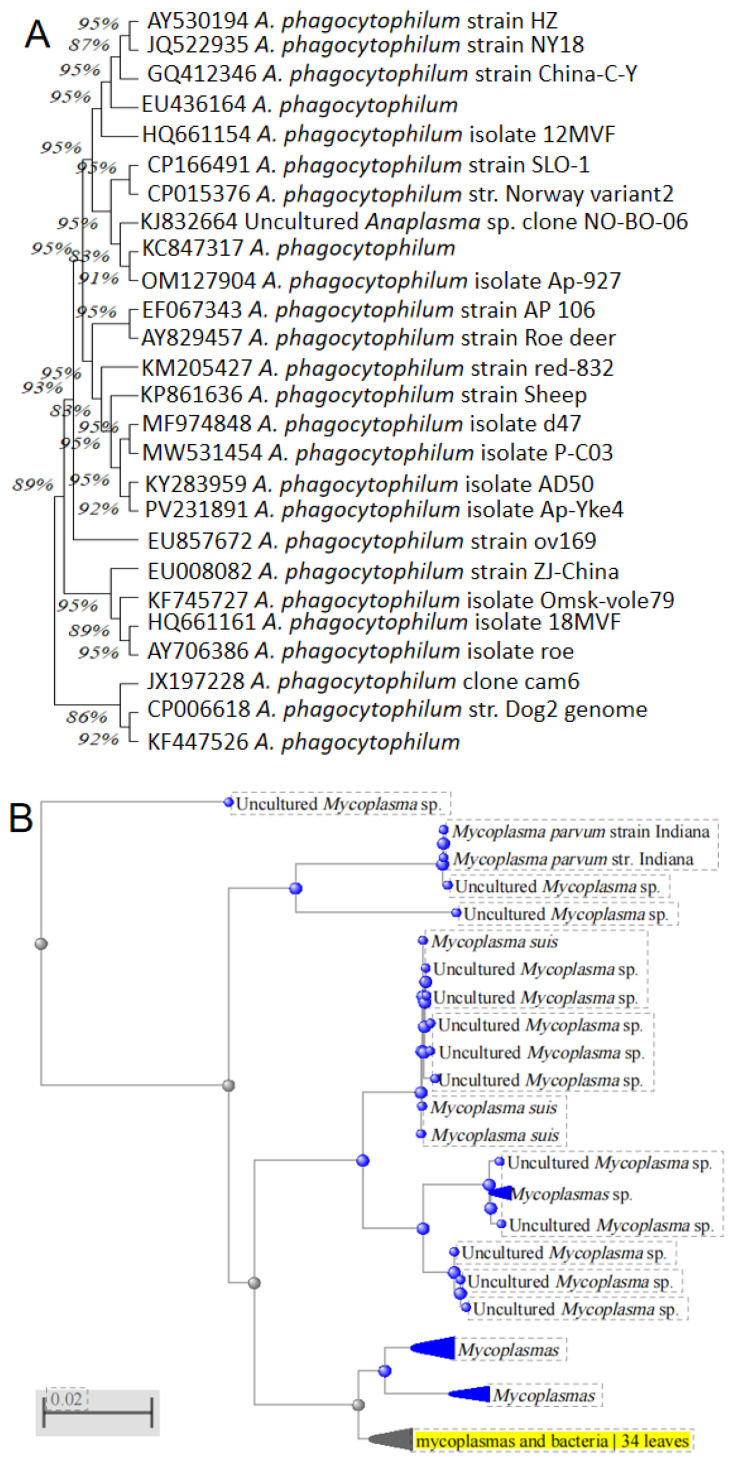
Phylogenetic tree based on the obtained key marker molecules for blood pathogens using the maximum likelihood method and the Tamura-Nei model with 1000 replicates (bootstrap tests). (**A**) system evolution tree based on the msp4 gene for *Anaplasma phagocytophilum*; (**B**) based on 23S ribosomal RNA gene for *Mycoplasma* sp. Note: Blue indicates clusters of *Mycoplasma* sp. from non-Gansu regions in NCBI databases. Yellow indicates clusters of *Mycoplasma* sp. on high sequence similarity to the sequences in this study.

To understand the genetic characteristics of prevalent strains in Gansu Province, we conducted gene sequencing and phylogenetic analysis on positive samples. Calculated haplotype diversity (Hd) and nucleotide diversity (π) for all populations using DnaSP v6. Added branch support values (bootstrap ≥ 90% or posterior probability ≥ 0.95) to all phylogenetic nodes. *Anaplasma ovis*: Analysis based on the Msp4 gene sequence revealed extremely low nucleotide diversity (π = 0.0008 ± 0.0001), among Gansu isolates. Compared to reference strains from Turkey (PP625124), Tunisia (MH292897), Egypt (OP244842), Sudan (KU497700), Mongolia (LC141078), Serbia (GQ925810), Cyprus (FJ460443), Croatia (PV626511), Italy (GQ130291), Malaysia (ON458035), and Russia (MT062868), the average nucleotide sequence identity reached 99.48%. Phylogenetic trees showed that Gansu isolates clustered closely with strains from these countries on the same evolutionary branch. *Theileria luwenshuni*: Analysis of the 18S rRNA sequences indicated that *Theileria luwenshuni* and *Theileria uilenbergi* were the primary species infecting sheep across different regions of Gansu. *T. luwenshuni* isolates from various locations shared identical taxonomic status, with 100% sequence identity. *Anaplasma phagocytophilum*: Genetic analysis of two isolates from the Baiyin region showed sequence identities ranging from 99.53% to 99.84% compared to strains from other countries and regions, also demonstrating high genetic conservation (Figure 3A). *Mycoplasma* spp.: Despite limited distribution in Gansu (≈3% prevalence, confined to Gannan, Wuwei, and Baiyin), these pathogens exhibited High haplotype diversity (Hd = 0.872) with 12 novel haplotypes (Figure 3B).

## 4. Discussion

As a large-scale molecular epidemiological surveillance of five major blood-borne pathogens in Gansu cattle herds, this study prioritizes the characterization of regional pathogen genetic diversity. Our results demonstrate a high degree of geographical specificity in pathogen distribution, jointly shaped by local ecological conditions, vector dynamics, and strain-specific genetic signatures. While the absence of recombination or mutation-focused analyses precludes direct mechanistic inferences, this baseline investigation establishes critical foundational data for future longitudinal studies and targeted interventions.

Compared to prior regional surveys in Northwest China covering 3 counties [10], and limited to *Anaplasma* spp. [17], this study establishes the most complete provincial dataset to date through surveillance across 12 counties in all 5 Gansu ecoregions, concurrently profiling five blood-borne pathogens via multi-locus genotyping (*n* = 254 strains, 2 genetic markers). The core finding reveals striking spatial heterogeneity in pathogen distribution—characterized by a complementary dominance pattern, though these observed variations represent associations rather than causation, necessitating future studies tracking vector densities, microclimate data, and livestock movement to test underlying hypotheses: *Theileria* spp. and *Anaplasma ovis* prevail province-wide, yet exhibit mutually exclusive high-prevalence zones. In Gannan Prefecture—a key pastoral region—we observed: Hyperendemic *A. ovis* (82.5%), *A. capra* co-infection (42.3%, with 24.7% dual *Anaplasma* infections). This distinct epidemiology aligns with local eco-vector dynamics reported in the literature: The cool-humid climate and dense vegetation of Gannan provide optimal habitats for ixodid ticks [18], potentially facilitating pathogen circulation and co-infection complexity among sheep. Spring infection peaks coincided with seasonal vector activity surges, suggesting a broader than previously recognized role of climate-mediated vector ecology in transmission dynamics. Furthermore, the observed geographical disparities likely reflect differences in vector ecology [19], although vector species were not characterized in this study.

In contrast, *Theileria* spp. infections are widely distributed across the Hexi Corridor (Zhangye, Jiuquan), and central and eastern Gansu (Linxia, Zhuanglang, Longnan), with exceptionally high infection rates observed specifically in Zhagana and Zhuanglang (87.5% and 75.0%, respectively). *Theileria* spp. transmission also relies on tick vectors, but its dominant vector species may differ from those of *Anaplasma* [19]. This geographical disparity may be related to the species and distribution range of the vectors. Furthermore, we observed significant seasonal fluctuations in *Theileria* spp. infection rates, with spring rates (23–34%) being significantly higher than those in other seasons (around 12%). This phenomenon aligns closely with the seasonal patterns of tick activity. Spring, characterized by rising temperatures and suitable humidity, is the peak period for tick hatching and activity. Grazing sheep have increased opportunities to encounter infectious ticks, leading to higher infection rates [20,21]. This finding holds significant implications for developing targeted control measures, suggesting that intensifying tick control and prophylactic medication before spring is key to reducing the risk of piroplasmosis.

Notably, no target pathogens were detected in regions such as Huining, Jingtai, Maqu, Jingyuan, and Qinghuan. This may stem from several factors: firstly, the natural environment and climatic conditions in these areas may be unsuitable for the survival of relevant vectors; secondly, effective integrated pathogens control programs may have been implemented locally; thirdly, the sampling period might have coincided with a low-activity phase of the pathogens. The negative result in Maqu, particularly as part of Gannan Prefecture, presents a stark contrast to the high positivity rates in other Gannan areas, meriting further investigation into specific reasons (e.g., altitude, microenvironment, differences in herd management practices).

While this study provides broad epidemiological insights, prevalence estimates for rare pathogens (e.g., *A. phagocytophilum*, *Mycoplasma* spp.) should be interpreted cautiously due to small positive counts. Estimates with small sample sizes (*n* ≤ 25) are reported as tentative observations due to limited statistical robustness (e.g., Jinchang, Longnan, Zhuanglang). Future surveillance with larger samples is needed to confirm these preliminary observations.

Our molecular evolutionary analysis revealed important genetic insights. Sequence analysis based on the Msp4 gene showed that *A. ovis* isolates prevalent in Gansu Province share extremely high genetic identity (average 99.48%) with strains from multiple countries across Europe, Asia, and Africa. This indicates that the Msp4 gene is highly conserved in *A. ovis*, consistent with previous research conclusions [22,23]. This high degree of conservation suggests that molecular detection tools (e.g., PCR) developed for this gene have broad application potential across different regions, while this PCR-based surveillance identifies spatial-temporal prevalence patterns, mechanisms underlying these patterns remain hypothetical and require future validation through integrated vector/environmental sampling. The associations suggested below represent plausible interpretations drawn from published ecological and molecular studies rather than direct evidence from this dataset. The close clustering of Gansu *A. ovis* strains with international isolates suggests phylogenetic linkages compatible with gene flow pathways proposed in other studies (e.g., livestock trade [24,25]), or migratory birds carrying vectors, and aligns with observed regional prevalence heterogeneity—suggesting trade routes may contribute to pathogen dispersal, though validation remains required. Similarly, sequences of *A*. *phagocytophilum* also exhibited high conservation (99.53–99.84% identity). As an important zoonotic pathogen, the low detection rate of *A. phagocytophilum* in Baiyin (0.7%) still warrants high vigilance due to its potential threat to public health security [26].

The high proportion of *A. ovis* and *A. capra* co-infection observed in Gannan (24.7%) is a noteworthy phenomenon. Co-infection may exacerbate the clinical condition of the host, leading to more severe anemia and emaciation, thereby causing greater economic losses to livestock production [27]. Furthermore, co-infection poses challenges for laboratory diagnosis and control, requiring detection methods capable of accurately differentiating and identifying the co-infecting pathogens.

This study has several limitations, including an unbalanced distribution of sampling sites and sample sizes across the province (e.g., Jinchang’s smaller sample size), a reliance solely on PCR for pathogen screening without subsequent isolation, culture, or genotyping to reveal genetic diversity, and a lack of concurrent vector/environmental data collection which constrains causal inferences about ecological drivers, transmission pathways, or vector dynamics; therefore, future research should increase sample sizes with more uniform site distribution, integrate vector surveys, environmental analysis, and whole-genome sequencing of pathogens to establish a systematic understanding of transmission dynamics and molecular epidemiology of blood-borne pathogens in Gansu livestock under regional surveillance constraints and validate observed epidemiological patterns.

## 5. Conclusions

This study establishes the first molecular baseline surveillance for *A. ovis* and *A. phagocytophilum* in Gansu Province, reporting preliminary genetic profiles and prevalence patterns in local sheep. While observed genetic diversity appears limited in sampled strains and regional/seasonal trends (e.g., elevated *Anaplasma* prevalence in Gannan, spring peaks for *Theileria*) suggest testable hypotheses about ecological drivers, these findings represent foundational surveillance data—not definitive evidence of evolutionary or epidemiological dynamics. Future whole-genome sequencing and environmental sampling are required to validate potential transmission pathways or genetic stability. This work prioritizes targets for control strategies but necessitates expanded monitoring before policy implementation.

## Figures and Tables

**Table 1:** Primers used in this study. All the primers used for the pathogens were designed as nested primers. The list contained the target gene, primer name, amplification length and primer source.

Pathogens	Target Gene	Primers	Sequences (5′–3′)	Amplification Length (bp)	Primer Source
*Mycoplasma* spp.	16s rRNA	A1 A2 B1 B2	GGATAGCAGCCCGAAAGG GCAGCCCAAGGCATAAGG CTACGGGAAGCAGCAGTG CTCGACCTAACATCAAATACCT	1060 506	Sun et al., 2006 [13]
*A. phagocytophilum*	Msp4	EEI EE2 SSAP2-F SSAP2-R	TCCTGGCTCAGAACGAACGCTGGCGGC GTCACTGACCCAACCTTAAATGGCTG GCTGAATGTGGGGATAATTTAT ATGGCTGCTTCCTTTCGGTTA	1430 641	Kawahara et al., 2006 [14]
*Anaplasma ovis*	Msp4	AMO-F AMO-R MSP4-F MSP4-R	GCTCCCTACTTGTTAGTGG TTAGCTGAACAGGAATCTTG CAAGCAGAGAGACCTCGTAT GGCTTTTGCTTCTCCGGG	795 584	Yan et al., 2006 [15]
*Theileria* spp.	18s rRNA	18srDNA-FI 18srDNA-RI 18srDNA-F2 18srDNA-R2	GATAACCGTGCTAATTGTAGG ATCGTCTTCGATCCCCTAACT AATTGTAGGGCTAATACATGTTCG GAAAACATCCTTGGCAAATGCTTTCGC	843 750	Mohamed et al., 2006 [16]

**Table 2:** The detection results of blood pathogens in different regions of Gansu Province.

Isolated Area	Sample No.	*Anaplasma ovis* (Bacterial Rickettsia) (%)	*Theileria* spp. (Apicomplexan Parasite) (%)	*Mycoplasma* spp. (Bacteria) (%)	*A. phagocytophilum* (Bacterial Rickettsia) (%)
Zhangye	40	27.5 (*n* = 11)	45.0 (*n* = 18)	/	/
Jinchang	5	/	*n* = 2	/	/
Tianshui	20	*n* = 5	*n* = 14	/	/
Jiuquan	109	/	9.40 (*n* = 43)	/	/
Linxia	76	/	43.40 (*n* = 33)	/	/
Gannan	80	24.70 (*n* = 24)	/	2.10 (*n* = 2)	/
Dingxi	180	4.40 (*n* = 8)	8.30 (*n* = 15)	/	/
Wuwei	96	2.10 (*n* = 2)	13.50 (*n* = 13)	3.13 (*n* = 3)	5.21 (*n* = 5)
Qingyang	100	/	14.00 (*n* = 14)	/	/
Pingliang	130	13.80 (*n* = 18)	/	/	/
Baiyin	287	4.20 (*n* = 12)	0.70 (*n* = 2)	3.80 (*n* = 11)	0.70 (*n* = 2)
Zhagana	24	/	*n* = 21	/	/
Zhuanglang	16	/	*n* = 12	/	/
Longnan	15	/	*n* = 7	/	/

Note: The diagonal line indicates that the blood pathogens corresponding to the horizontal heading was not detected in the sample. All inter-county prevalence comparisons now restricted to counties with *n* ≥ 30 samples (validated by χ^2^-test power analysis). Counties with *n* < 30 are flagged in tables and solely reported for pathogen detection (yes/no), not prevalence rates. Huining, Jingtai, Maqu, Jingyuan, Qinghuan: No pathogens detected (*n* = 345).

## Data Availability

The data that support the findings of this study are available upon reasonable request from the authors.
